# High-performance bilayer flexible resistive random access memory based on low-temperature thermal atomic layer deposition

**DOI:** 10.1186/1556-276X-8-92

**Published:** 2013-02-19

**Authors:** Run-Chen Fang, Qing-Qing Sun, Peng Zhou, Wen Yang, Peng-Fei Wang, David Wei Zhang

**Affiliations:** 1 State Key Laboratory of ASIC and System, Department of Microelectronics, Fudan University, 200433, Shanghai, China

**Keywords:** Flexible memory, Atomic layer deposition, Low temperature

## Abstract

We demonstrated a flexible resistive random access memory device through a low-temperature atomic layer deposition process. The device is composed of an HfO_2_/Al_2_O_3_-based functional stack on an indium tin oxide-coated polyethylene terephthalate substrate. After the initial *reset* operation, the device exhibits a typical bipolar, reliable, and reproducible resistive switching behavior. After a 10^4^-s retention time, the memory window of the device is still in accordance with excellent thermal stability, and a 10-year usage is still possible with the resistance ratio larger than 10 at room temperature and at 85°C. In addition, the operation speed of the device was estimated to be 500 ns for the *reset* operation and 800 ns for the *set* operation, which is fast enough for the usage of the memories in flexible circuits. Considering the excellent performance of the device fabricated by low-temperature atomic layer deposition, the process may promote the potential applications of oxide-based resistive random access memory in flexible integrated circuits.

## Background

Since flexible electronic system (FES) appeals to be light, convenient, has conformal contingence with the crooked surface, and excellent interfaces with humans, it ought to be a prospective existing form of electronic product to substitute its clumsy predecessors manufactured and packaged by traditional bulk silicon technology [1,2]. Up to now, multifarious electronic components, such as integrated circuits (ICs) [3,4], active matrix organic light-emitting diodes [5], sensors [6], radiofrequency identification antennas [7], and solar cells [8,9], have been fabricated on flexible substrates and are delved by many researchers. As we know, among all the components used in ICs, good and reliable memories [10,11] will maximize the functionality of ICs, and it is also important for the FES.

Among all the memories, nonvolatile resistive random access memory (RRAM) is the most promising candidate because of its low power consumption, high speed, simple structure, and high packaging density, compared with its counterparts such as flash memory and DRAM [12-14]. Currently, oxides, such as STO [15], HfO_2_[16], NiO [17], Al_2_O_3_[18], ZnO [19], and GO [20], have received much interest in resistive switching research. Among the oxides mentioned, HfO_2_ has been profoundly studied and contains great potentiality to be put into applications. However, the application of HfO_2_-based RRAM on flexible substrate is still rare.

In recent years, atomic layer deposition (ALD) has emerged as a new technique for depositing films, particularly for fabricating oxide films. Owing to its self-limiting mechanism during the process, excellent step coverage and conformal thickness of the film can be achieved [21]. Although the deposition of oxide film by ALD on bulk silicon is very mature, seldom had researchers used this method to deposit films on flexible substrate. The main reason is that the flexible substrate could not undergo high-temperature processing above 200°C, except in some cases such as depositing films using plasma-enhanced atomic layer deposition under low temperature where plasma damage and degradation of the step coverage is unavoidable [22].

In this letter, we fabricated a bilayer flexible RRAM device based on HfO_2_/Al_2_O_3_ films under low temperature, with resistive layers deposited using a low-temperature ALD process at 120°C and the electrodes sputtered by direct current (DC) magnetron reactive sputtering at room temperature. The devices fabricated by these methods exhibit impressive resistive switching characteristics with reliable data retention properties under room temperature and elevated temperature up to 85°C.

## Methods

Flexible RRAM was fabricated on polyethylene terephthalate (PET) substrate coated by indium tin oxide (ITO) conducting film, and ITO serves as the bottom electrode in our devices. During the process, the substrate was fixed on a 3-in wafer with polyimide tapes in order to maintain sufficient mechanical support. The Al_2_O_3_ layer was deposited by 41 cycles of low-temperature ALD at 120°C with trimethyl aluminum (TMA) and water as precursors. Subsequently, the HfO_2_ layer was deposited by 67 cycles within the same framework using tetrakis(ethylmethylamino)hafnium (TEMAH) and water as precursors. TMA was pulsed at room temperature, and TEMAH was heated to 85°C to offer enough evaporation pressure. Al_2_O_3_ film was deposited with a pulse time of 0.1 and 0.2 s for TMA and water, and the purging time for TMA and water was 5 and 20 s, respectively. The deposition method of HfO_2_ was derived from our previous work [23]. Finally, a 50-nm TiN top electrode was sputtered on the resistive layer by DC magnetron reactive sputtering through a metal shadow mask with a diameter of 400 μm.

The thicknesses of the HfO_2_ and the Al_2_O_3_ layer were estimated to be 10.1 and 4.9 nm by Sopra GES5E spectroscopic ellipsometry. X-ray photoelectron spectroscopy (XPS) of HfO_2_ and Al_2_O_3_ on the PET substrate was performed using a Kratos Axis Ultra DLD XPS (Kratos Analytical, Ltd., Manchester, UK). Electrical properties at room temperature and at 85°C of the device were assayed using an Agilent B1500A (Agilent Technologies, Inc., Santa Clara, CA, USA) semiconductor parameter analyzer and an Agilent B1525A high-voltage semiconductor pulse generator. Impedance of high and low resistance states was analyzed by an Agilent 4294A precision impedance analyzer. The device was tested with top biased and grounded bottom electrodes.

## Results and discussion

**Figure 1:**
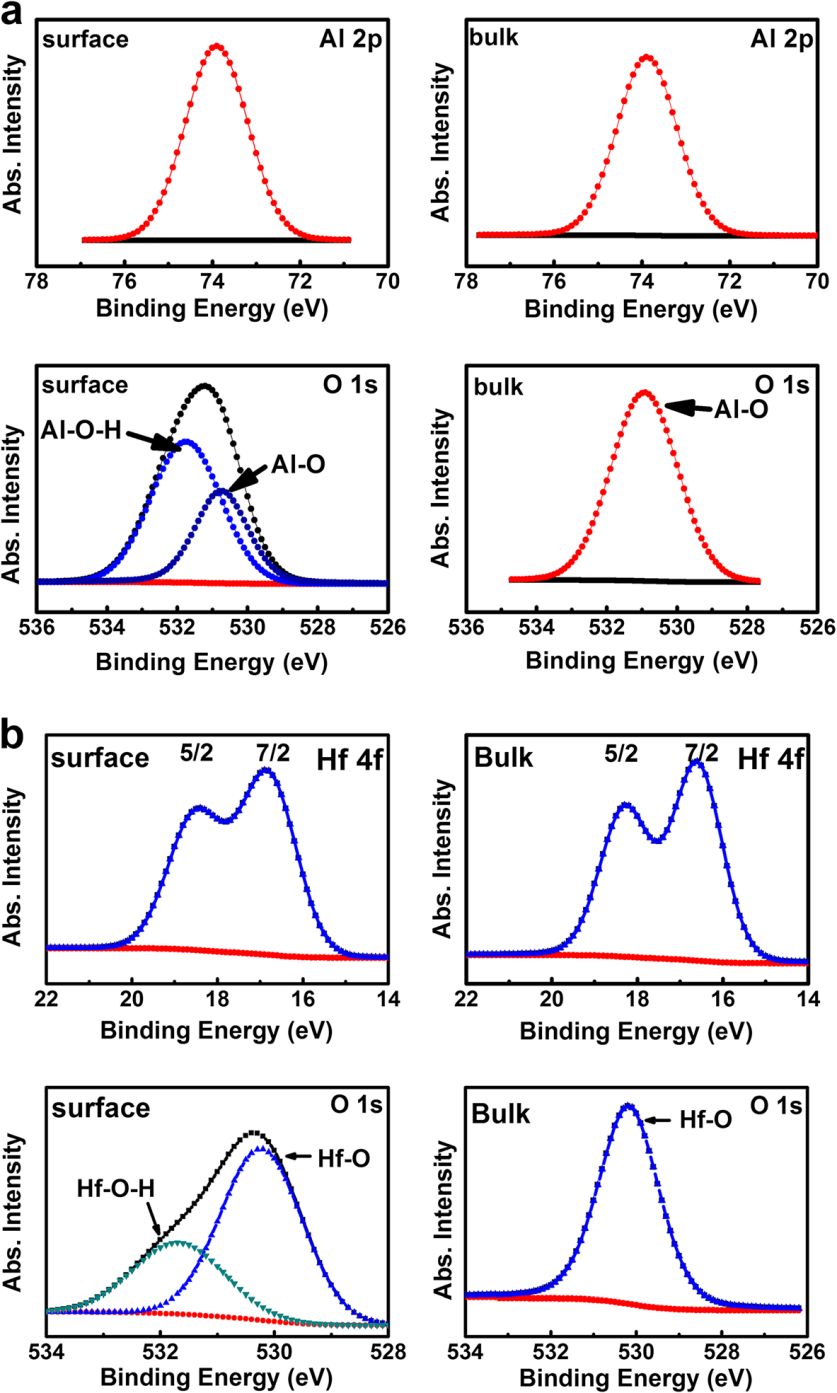
**The XPS spectra.** (**a**) Al 2*p* and O 1*s* peaks at the surface and in the bulk of the Al_2_O_3_ film. (**b**) Hf 4*f* and O 1*s* peaks at the surface and in the bulk of HfO_2_ film.

The XPS spectra of HfO_2_ and Al_2_O_3_ films are respectively shown in Figure 1a,b. In Figure 1a, the binding energies of Al 2*p* in the bulk and at the surface of the Al_2_O_3_ film are both at 73.9 eV, and the binding energies of O 1*s* in the bulk and at surface of the Al_2_O_3_ film show that the Al-O bond is at about 530.8 eV without any shifts. In Figure 1b, the bulk and surface XPS spectra of the HfO_2_ film illustrate that the binding energies of the Hf 4f^5/2^ and 4f^7/2^ are at the positions of about 18.4 and 16.7 eV, respectively, with a 1.7-eV spin-orbit splitting. From the O 1*s* spectrum in Figure 1b, the Hf-O bond is at 530 eV in the interior and at the surface of the HfO_2_ film [24]. However, from the surface XPS of O 1*s* in both Al_2_O_3_ and HfO_2_, the existence of -OH is observed with a peak at around 532 eV. This is either incorporated by residue water precursors during the process because of the high desorption energy of water at low temperatures or exposing the film to the atmosphere (CO_2_ and moisture) before XPS measurement [23]. The XPS qualification report shows that the ratios of the O/Al in the bulk of the Al_2_O_3_ film and the O/Hf in the bulk of the HfO_2_ are about 1.7 and 2, respectively, which means that our films obtained at low temperature are almost stoichiometric.

**Figure 2:**
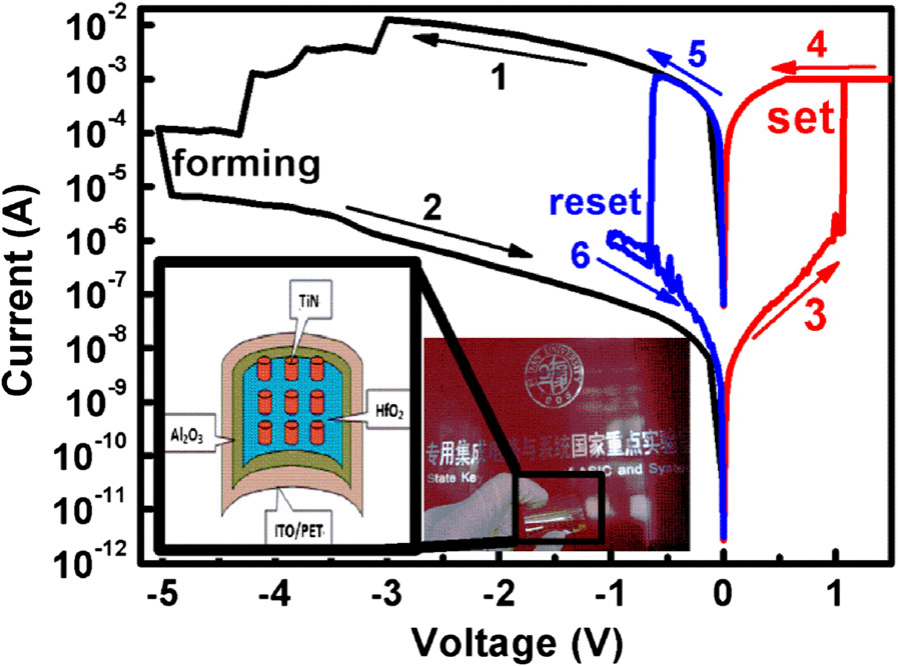
**The initial *reset *operation and the typical resistive switching characteristics of the flexible RRAM.** Inset: the photograph and schematic structure of the device.

Typical *I*-*V* characteristics of the device are shown in Figure 2, which indicates a bipolar resistive switching. The initial resistance state of the TiN/HfO_2_/Al_2_O_3_/ITO flexible RRAM (schematically shown in the inset of Figure 2) device was found (curve 1) to be even lower than the low resistance state (LRS) of the device, and an excess negative voltage was applied to reset the device to high resistance state (HRS). The initial reset voltage and current were −3 V and 10 mA, respectively. This phenomenon was not observed in RRAMs grown at high temperatures, except in some cases after high-temperature annealing [25-27]. We attribute this phenomenon to the high density of defects in the film grown at low temperature. As with our low-temperature ALD processing using H_2_O as oxidant, it is inevitable that there will be some incomplete reactions during the process, such as residual -OH groups, fixed positive charges, and oxygen vacancies. It is considered that when the density of defects exceeds the percolation theory threshold value, the resistance of the insulating layer will be lower than the typical value [26,28]. This large density of defects may be very suitable for RRAM applications which work dependently on the defects. After the initial *reset* operation, the *set* operation was achieved by sweeping a positive voltage from 0 to 1.5 V with 1 mA of current compliance to protect the device from a hard breakdown (curve 3). An abrupt increase of current was observed at 1 V, and the device was set to LRS (approximately 650 Ω). A negative bias was then applied to the device by a sweep from 0 to −1 V, and a sudden descent of current occurred at −0.6 V, indicating that the device was reset to HRS with a reset current in the same magnitude as the set current.

**Figure 3:**
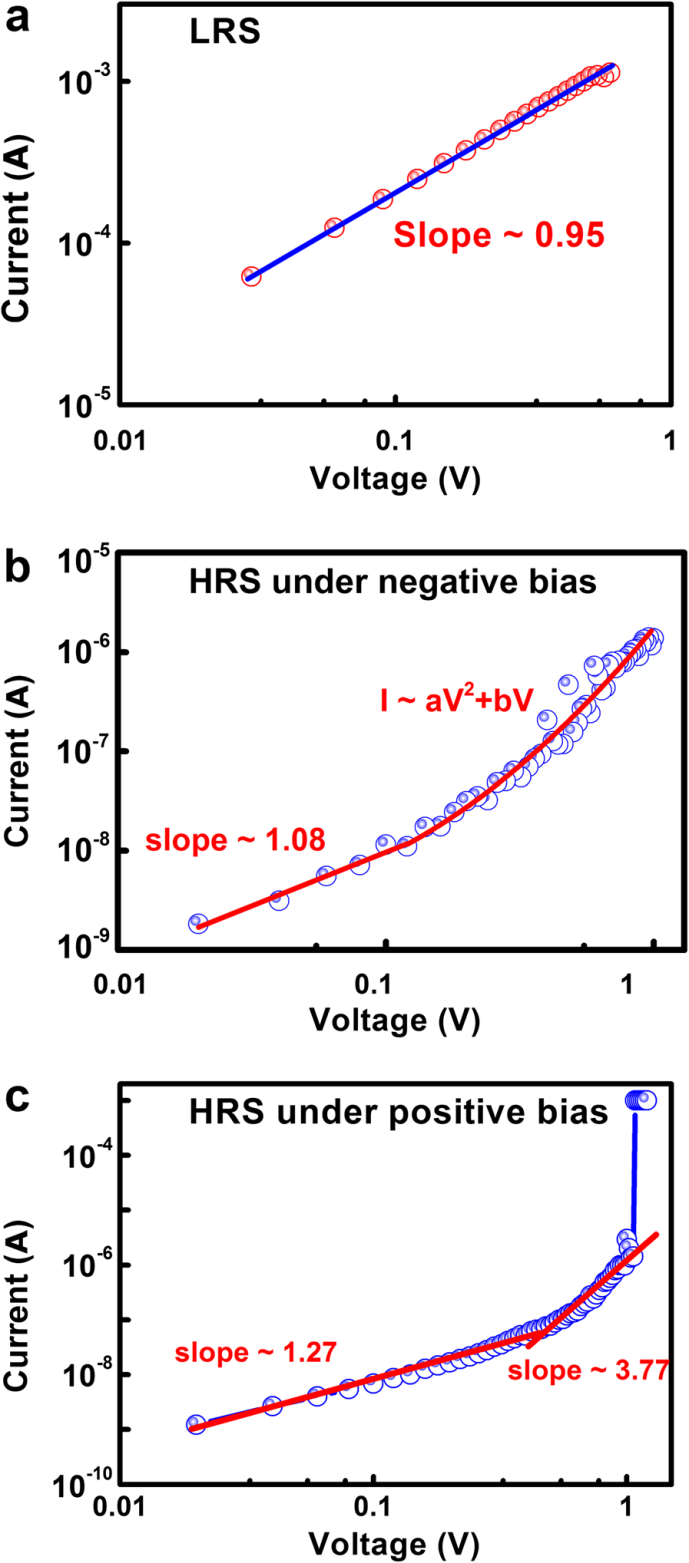
**Dual logarithmic plots of the current–voltage characteristics.** (**a**) ON state device, (**b**) OFF state device under negative bias, and (**c**) OFF state device under positive bias.

To further investigate the conduction mechanism in the flexible RRAM, the *I*-*V* curves of the ON and OFF states were re-plotted in a dual logarithmic plot. As shown in Figure 3a, the logarithmic plot and linear fitting of the previous *I*-*V* curve for the device in LRS show a typical ohmic conduction with a slope of 0.95, which is considered to be the formation of conductive filaments in the memory cell during the *set* process. On the other hand, the conduction mechanism of the device in HRS seems to be more complicated, with considerable disparities in negative and positive sweepings. The fitting result for the device in HRS under negative bias is presented in Figure 3b, and the slopes of the curve differ from each other under different voltages. When the electric field is small, the *I*-*V* slope is about 1.08, which conforms to ohmic conduction. However, when the voltage enters into the high electric field, the relationship between logarithm voltage and logarithm current turns to be an aV^2^ + bV relation, which is the classical space charge-limited conduction (SCLC). However, for the conduction behavior of the OFF state in devices under positive bias (Figure 3c), the slope is estimated to be 1.27 when the electric field is small, and the slope raises to 3.77 when the electric field is large enough until it approaches the compliance current (1 mA). As it is widely accepted that in oxide-based films the electron hops across the film through the body oxygen vacancies or defects, we attribute the conduction mechanism for the device in HRS under positive bias to be the trap-assisted tunneling (TAT) conduction [29]. When a negative bias was applied on the device, electrons are injected from the top electrode (TE) to the oxide and then proceed to the bottom electrode (BE). The resistance of TE to oxide is much larger than that of oxide to BE. As a result, the current is limited by the available electron in the oxide and leads to SCLC conduction. On the other hand, when a positive voltage was applied on the device, electrons are injected from BE to the oxide and then proceed to the TE. The current is limited by the traps available in the oxide near TE. As a result, the conduction mechanism will possibly be TAT.

**Figure 4:**
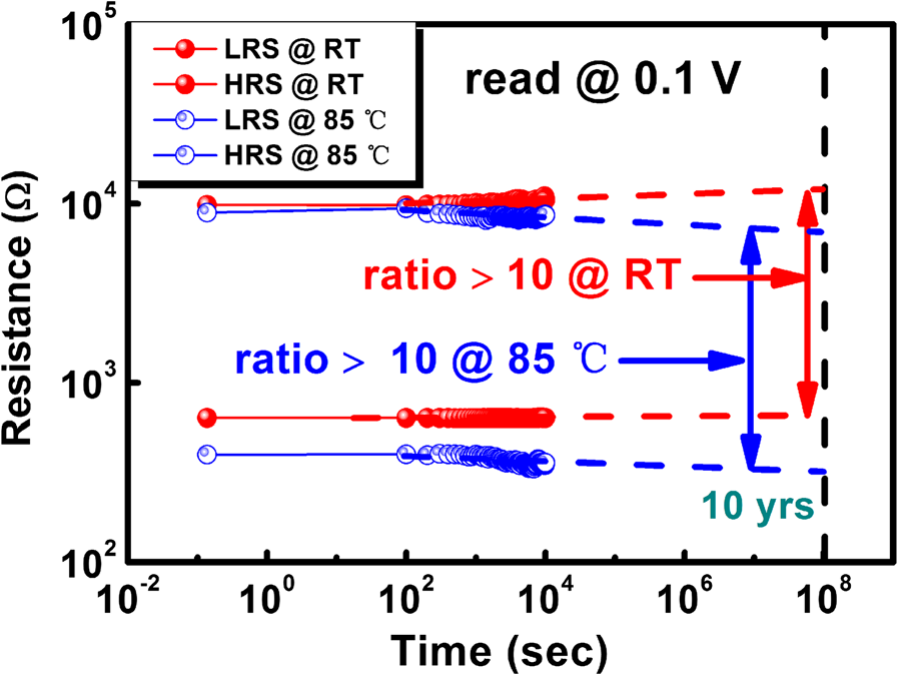
**Read disturbance test for device after 10**^**4**^-**s retention time under room temperature and at 85°C.** No significant degradation of resistance ratio was observed under room temperature, and there is a slightly parallel descent of the HRS and LRS at 85°C.

Figure 4 shows the data retention characteristics of the flexible RRAM device at room temperature and under high temperature up to 85°C. Both HRS and LRS were read at 0.1 V for 10^4^ s, and a predetermination of the long-term retention was made. At room temperature, no significant degradation of the memory window was observed, with the HRS ascending slightly. It suggests that sufficient memory margin still exists when the device undergoes decade employment. At elevated temperature (85°C), even with descents of both LRS and HRS, the memory window is still in accordance with excellent thermal stability, and a 10-year usage is still possible, with the resistance ratio larger than 10.

**Figure 5:**
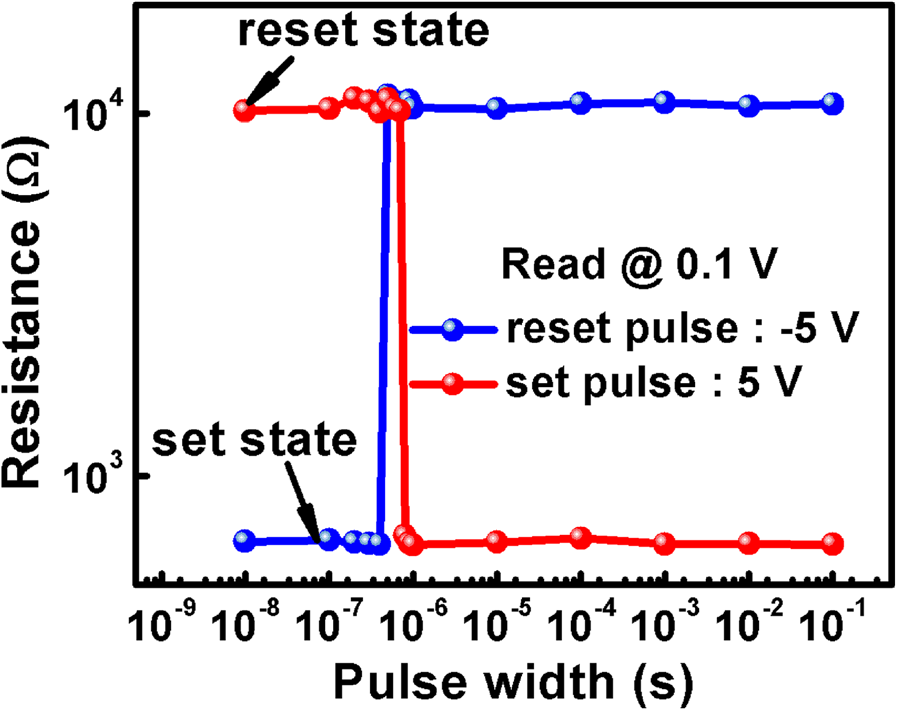
**The behavior of the TiN/HfO**_**2**_**/Al**_**2**_**O**_**3**_**/ITO/PET memory cell under different pulses.** HRS and LRS are read at 0.1 V, and the *set* and *reset* operations of the devices were achieved with different pulsing widths at ±5 V.

The speed of the *set* and *reset* operations with different pulse widths at ±5 V is exhibited in Figure 5, and the resistance state of the device after the pulse was read at 0.1 V. We found that the resistive switching phenomenon occurs when the pulse width is larger than 500 ns for *reset* operation and 800 ns for *set* operation. The operation speed of the memory cell is a little faster than some cases before [22,30].

**Figure 6:**
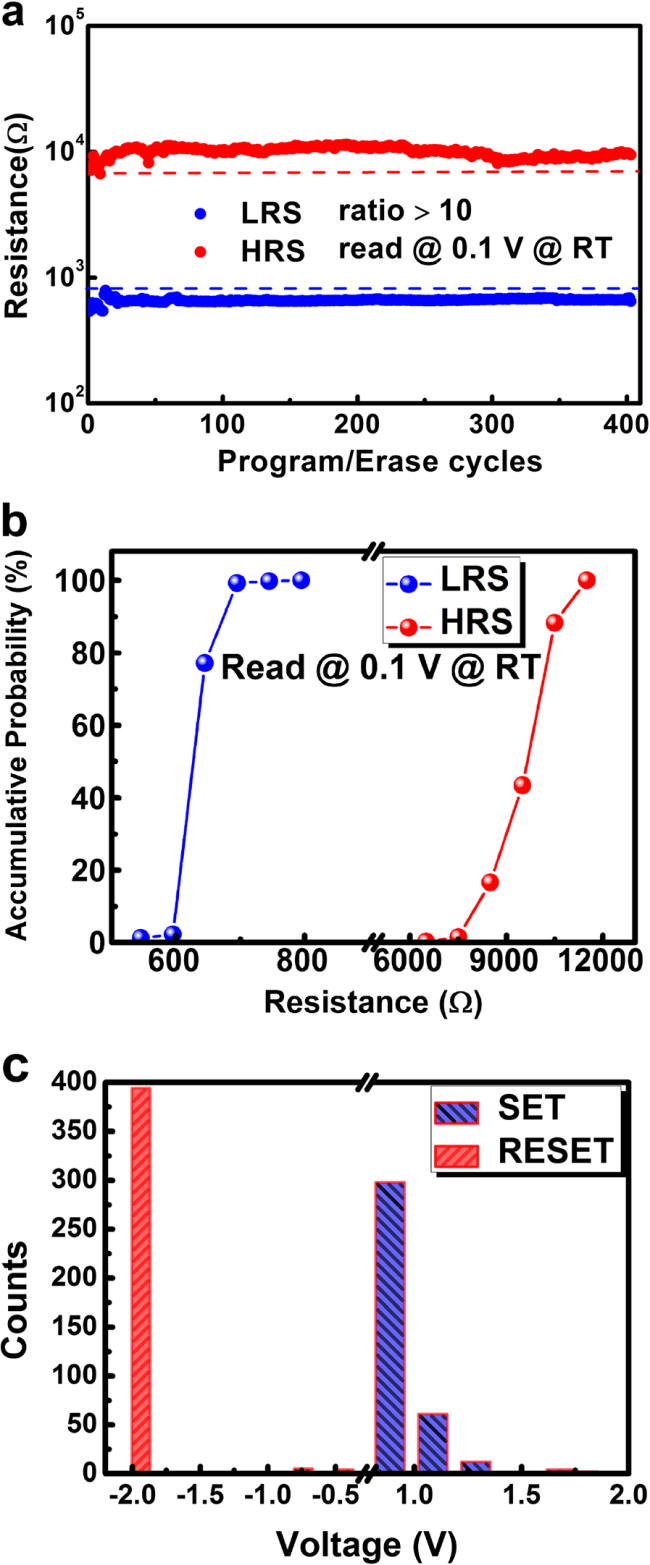
**The DC endurance test of the device.** Voltage sweeping was from 0 V to 2 V for *set* and from 0 V to −2 V for *reset* at room temperature, with a reading voltage of 0.1 V. (**a**) The continuous program and erase test, (**b**) the statistical result of the *set* and *reset* voltages, and (**c**) the statistical result of the resistance distributions of the LRS and HRS.

Stable and reproducible switching characteristics have been displayed in Figure 6 with a consistent 400 switching cycle without failures by DC sweeping. The sweeping voltage was applied from 0 to 2 V for *set* and 0 to −2 V for *reset* with a reading voltage of 0.1 V at room temperature. In Figure 6a, the result of the endurance test shows that memory ratio remains above 10:1 all along. Furthermore, statistics of the resistances and operation voltages are conducted separately according to the endurance test result. The resistance distributions of the LRS and HRS have been shown in Figure 6b, and we can find that only a small dispersion, with almost 90% of the LRS around 0.6 kΩ and 80% of the HRS around 10 kΩ, existed during the switching. In addition, Figure 6c shows the operation voltage distributions for *set* and *reset*. It can be obviously observed that almost 99% of the *reset* voltages are near −2 V and almost 85% of the *set* voltages are around 1 V. Through all the statistical results and previous test result, we can conclude that our flexible RRAM is characterized with high uniformity and reliability.

**Figure 7:**
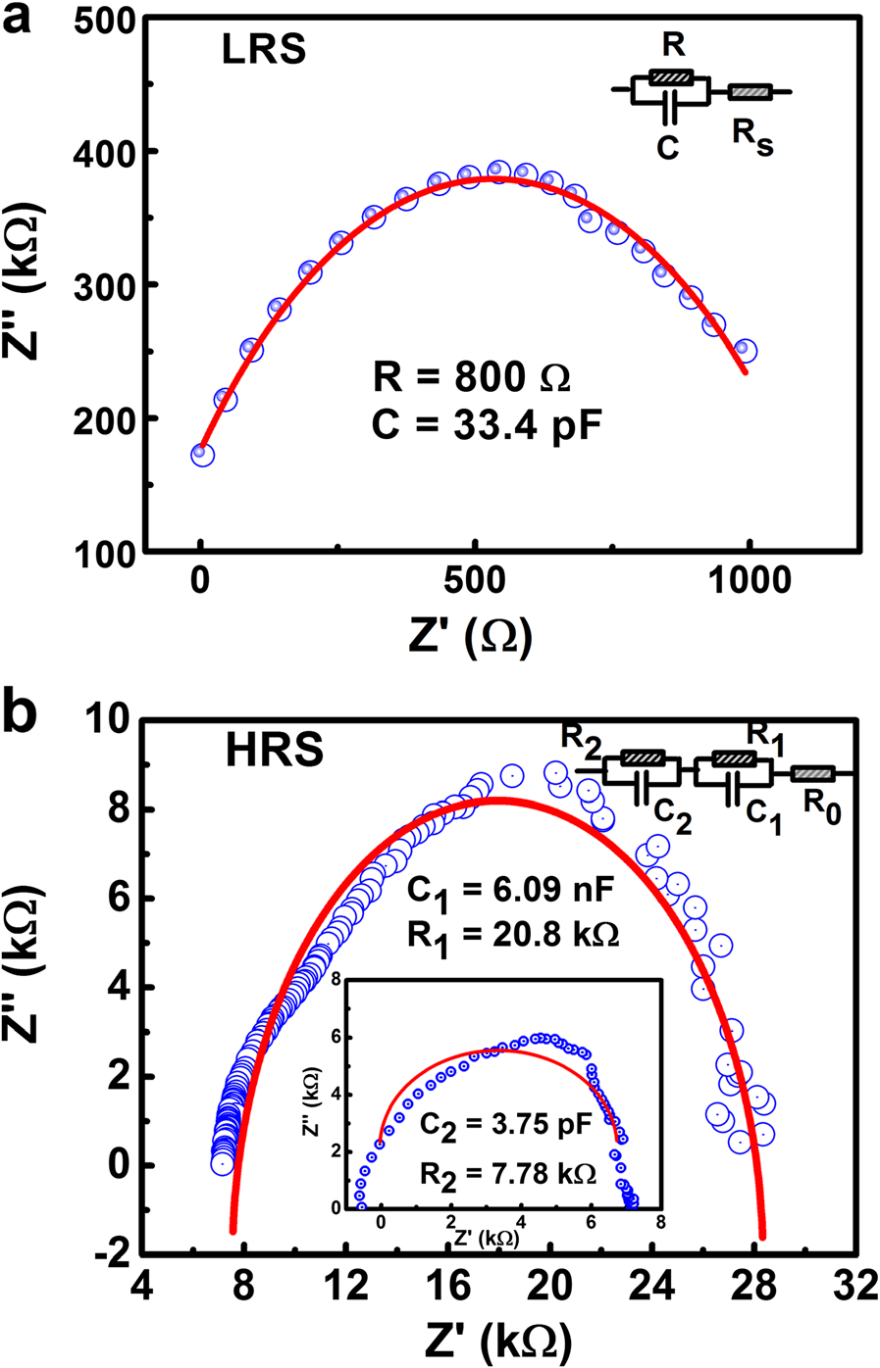
**The Nyquist plots.** (**a**) LRS and (**b**) HRS from impedance measurements. Their fittings to the equivalent circuits (solid line) and the circuit models as well as their parameters were also presented.

To inspect the equivalent circuit model of the device, we measured the impedance of the device in HRS and LRS in the *Z*-*Z* (*θ*) mode by applying 20 mV of AC small signal (40 Hz to 110 MHz) to the device. Figure 7 shows the Nyquist plot (*Z*″-*Z*^′^, *Z*″, and *Z*^′^ represent the absolute value of imaginary parts and real parts of the impedance) of the device in the LRS and HRS. In Figure 7a, one semicircle is observed in the LRS, indicating the equivalent RC parallel circuit model. Parameters from the fitting results reveal the existence of a tiny capacitance and a big resistance, which is in consonance with the conductive filament (CF) theory that when the RRAM is in LRS, it is mainly a resistance formed by the CF [10]. On the other hand, the calculated parameters for the HRS are shown in the inset of Figure 7b, and the device exhibits two different semicircles which indicate the complex equivalent circuit model that contains two RC parallel sections in series. In the LRS of the device, conducting filaments are formed in the device, and as a result, the device can be considered as a resistor with small resistance and a capacitor (the area without formed filaments) with small capacitance. On the other hand, when the device is in HRS, conducting filaments are ruptured at a certain position in the oxide. The ruptured place will induce an additional tunneling resistor with big resistance and a capacitor with big capacitance.

## Conclusions

In conclusion, a highly reliable and uniform flexible RRAM based on the TiN/HfO_2_/Al_2_O_3_/ITO structure, fabricated by a low-temperature process, was investigated. The fresh cell shows an ultra-low resistance state, and after the initial *reset* operation, a typical bipolar reliable and reproducible resistive switching behavior was demonstrated. It is found that the memory window is still in accordance with excellent thermal stability after a 10^4^-s retention time, and a 10-year usage is still possible with the resistance ratio larger than 10 at room temperature and at 85°C. The resistance of the LRS and HRS exhibits a very concentrated distribution with almost 90% of the LRS around 0.6 kΩ and 80% of the HRS around 10 kΩ. The developed low-temperature process for the memories may promote the potential applications of oxide-based RRAM in flexible ICs.

## Competing interests

The authors declare that they have no competing interests.

## Authors' contributions

RCF carried out the sample fabrication and drafted the manuscript. WY carried out the device measurements. PZ and PFW participated in writing the manuscript and in the discussion of results. QQS and DWZ participated in the design of the study and performed statistical analysis. All authors read and approved the final manuscript.

## Authors' information

RCF received his B.S. degree in Physics and Electronics from Nanjing Information Engineering University, Nanjing, China in 2010. He is currently studying at the School of Microelectronics, Fudan University for his Master's degree. His research interests include flexible memory and device design. QQS received his B.S. degree in Physics, his M.S. and Ph.D. degrees in Microelectronics and Solid state Electronics from Fudan University, Shanghai, China in 2004 and 2009, respectively. He is currently an associate professor at the School of Microelectronics in Fudan University. His research interests include fabrication and characterization of advanced metal oxide semiconductor field effect transistors, mainly high-k dielectric-based devices. He is also interested in design, fabrication, and characterization of advanced memory devices, such as resistive switching memory devices and Flash. PZ received his B.S. degree in Physics and Ph.D. degree in Optics from Fudan University, Shanghai, China in 2000 and 2005, respectively. He is currently an associate professor at the School of Microelectronics, Fudan University. His research interests include fabrication and characterization of advanced metal oxide semiconductor field effect transistors, advanced memory devices, and graphene device. WY received her B.S. degree in Physics and Electronics from Henan University, Henan, China in 2010. She is currently studying at the School of Microelectronics, Fudan University for her Ph.D. degree. Her research interests include low-power circuit, memory and device design, and theoretical and experimental investigations of two dimensional materials. PFW received his B.S. and M.S. degrees from Fudan University, Shanghai, China in 1998 and 2001, respectively, and his Ph.D. degree from the Technical University of Munich, München, Germany in 2003. Until 2004, he was with the head of the Memory Division of Infineon Technologies in Germany on the development and process integration of novel memory devices. Since 2009, he has been a professor at Fudan University. His research interests include design and fabrication of semiconductor devices and development of semiconductor fabrication technologies such as high-k gate dielectrics and copper/low-k integration. DWZ received his B.S., M.S., and Ph.D. degrees in Electrical Engineering from Xi’an Jiaotong University, Xi’an, China in 1988, 1991, and 1995, respectively. In 1997, he was an associate professor at Fudan University, Shanghai, China, where he has been a full professor since 1999. He is currently the Dean of the Department of Microelectronics and the Director of the Fudan-Novellus Interconnect Research Center. He has authored more than 200 referred archival publications and is the holder of 15 patents. More than 50 students have received their M.S. or Ph.D. degrees under his supervision. His research interests include integrated-circuit processing and technology, such as copper interconnect technology, atomic layer deposition of high-k materials; semiconductor materials and thin-film technology; new structure dynamic random access memory (RAM), Flash memory, and resistive RAM; and metal oxide semiconductor FET based on nanowire and nanotube and tunneling FET.

## References

[B1] Reuss RH Chalamala BR Moussessian A Kane MG Kumar A Zhang DC Rogers JA Hatalis M Temple D Moddel G Eliasson BJ Estes MJ Kunze J Handy ES Harmon ES Salzman DB Woodall JM Alam MA Murthy JY Jacobsen SC Olivier M Markus D Campbell PM Snow E Macroelectronics: perspectives on technology and applications Proc IEEE 2005 8 1239 1256

[B2] Tsutsui T Fujita K The shift from “hard” to “soft” electronics Adv Mater 2002 8 949 952

[B3] Cao Q Kim HS Pimparkar N Kulkarni JP Wang CJ Shim M Roy K Alam MA Rogers JA Medium-scale carbon nanotube thin-film integrated circuits on flexible plastic substrates Nature 2008 8 495 500 10.1038/nature07110 18650920

[B4] Kim MG Kanatzidis MG Facchetti A Marks TJ Low-temperature fabrication of high-performance metal oxide thin-film electronics via combustion processing Nat Mater 2011 8 382 388 10.1038/nmat3011 21499311

[B5] Li JF Hu LB Liu J Wang L Marks TH George G Indium tin oxide modified transparent nanotube thin films as effective anodes for flexible organic light-emitting diodes Appl Phys Lett 2008 8 083306

[B6] Kuniharu T Toshitake T Johnny CH Hyunhyub K Andrew GG Paul WL Ronald SF Ali J Nanowire active-matrix circuitry for low-voltage macroscale artificial skin Nat Mater 2010 8 821 826 10.1038/nmat2835 20835235

[B7] Rutherglen C Jain D Burke P Nanotube electronics for radiofrequency applications Nat Nanotechnol 2009 8 811 819 19946283 10.1038/nnano.2009.355

[B8] Kaltenbrunner M White MS Glowacki ED Sekitani T Someya T Sariciftci NS Bauer S Ultrathin and lightweight organic solar cells with high flexibility Nat Commun 2012 8 1 7 10.1038/ncomms1772 PMC3337988 22473014

[B9] Galstyan V Vomiero A Concina I Braga A Brisotto M Bontempi E Faglia G Sberveglieri G Vertically aligned TiO_2_ nanotubes on plastic substrates for flexible solar cells Small 2011 8 2437 2442 10.1002/smll.201101356 21793205

[B10] Waser R Dittmann R Staikov G Szot K Redox-based resistive switching memories–nanoionic mechanisms, prospects, and challenges Adv Mater 2009 8 2632 2663 10.1002/adma.200900375 36751064

[B11] Strukov DB Snider GS Stewart DR Williams RS The missing memristor found Nature 2008 8 80 83 10.1038/nature06932 18451858

[B12] Sheu SS Cheng KH Chang MF Chiang PC Lin WP Lee HY Chen PS Chen YS Wu TY Chen FT Su KL Kao MJ Tsai MJ Fast-write resistive RAM (RRAM) for embedded applications IEEE Design & Test of Computers 2011 8 64 7123666128

[B13] Tseng YH Huang CE Kuo CH Chih YD King YC Lin CJ A new high-density and ultrasmall-cell-size contact RRAM (CR-RAM) with fully CMOS-logic-compatible technology and circuits IEEE Trans Electron Dev 2011 8 53 58

[B14] Sawa A Resistive switching in transition metal oxides Mater Today 2008 8 28 36

[B15] Szot K Speier W Bihlmayer G Waser R Switching the electrical resistance of individual dislocations in single-crystalline SrTiO_3_ Nat Mater 2006 8 312 320 10.1038/nmat1614 16565712

[B16] Chen YS Lee HY Chen PS Tsai CH Gu PY Wu TY Tsai KH Sheu SS Lin WP Lin CH Chiu PF Chen WS Chen FT Lien C Tsai MJ Challenges and opportunities for HfO_*x*_ based resistive random access memory IEEE International Electron Devices Meeting: 5–7 Dec. 2011 2011 Washington DC: Washington DC: IEEE 31.3.1 31.3.4

[B17] Sun QQ Gu JJ Chen L Zhou P Wang PF Ding SJ Zhang DW Controllable filament with electric field engineering for resistive switching uniformity IEEE Electron Device Lett 2011 8 1167 1169

[B18] Chen L Xu Y Sun QQ Liu H Gu JJ Ding SJ Zhang DW Highly uniform bipolar resistive switching with Al_2_O_3_ buffer layer in robust NbAlO-based RRAM IEEE Electron Device Lett 2010 8 356 358

[B19] Chang WY Lai YC Wu TB Wang SF Chen F Tsai MJ Unipolar resistive switching characteristics of ZnO thin films for nonvolatile memory applications Appl Phys Lett 2008 8 022110 10.1063/1.2834852

[B20] Wang LH Yang W Sun QQ Zhou P Lu HL Ding SJ Zhang DW The mechanism of the asymmetric SET and RESET speed of grapheme oxide based flexible resistive switching memories Appl Phys Lett 2012 8 063509 10.1063/1.3681366

[B21] George SM Atomic layer deposition: an overview Chem Rev 2010 8 111 131 10.1021/cr900056b 19947596

[B22] Kim SJ Kim SK Jeong HY Flexible memristive memory array on plastic substrates Nano Lett 2011 8 5438 5442 10.1021/nl203206h 22026616

[B23] Fang RC Wang LH Yang W Sun QQ Zhou P Wang PF Ding SJ Zhang DW Resistive switching of HfO_2_ based flexible memories fabricated by low temperature atomic layer deposition J Vac Sci Technol B 2012 8 020602

[B24] Moulder JF Stickle WF Sobol PE Bomben KD Chastain L Handbook of X-ray Photoelectron Spectroscopy 1992 Eden Prairie: Perkin Elmer

[B25] Son JY Kim CH Cho JH Shin YH Jang HM Self-formed exchange bias of switchable conducting filaments in NiO resistive random access memory capacitors ACS Nano 2010 8 3288 3292 10.1021/nn100323x 20433193

[B26] Chen YS Lee HY Chen PS Wu TY Wang CC Tzeng PJ Chen F Tsai MJ Lien C An ultrathin forming-free HfO_*x*_ resistance memory with excellent electrical performance IEEE Electron Device Lett 2010 8 1473 1475

[B27] Chien WC Chen YC Lee FM Lin YY Lai EK Yao YD Gong J Horng SF Yeh CW Tsai SC Lee CH Huang YK Chen CF Kao HF Shih YH Hsieh KY Lu CY A novel Ni/WO_*x*_/W resistive random access memory with excellent retention and low switching current Jpn J Appl Phys 2011 8 04DD11 10.1143/JJAP.50.04DD11

[B28] Zhao CZ Zhang JF Zahid MB Efthymiou E Lu Y Hall S Peaker AR Groeseneken G Pantisano L Degraeve R Gendt SD Heyns M Hydrogen induced positive charge in Hf-based dielectrics Microelectronic Engineering 2007 8 2354 2357 10.1016/j.mee.2007.04.096

[B29] Yu SM Guan XM Wong HS Conduction mechanism of TiN/HfO_*x*_/Pt resistive switching memory: a trap-assisted-tunneling model Appl Phys Lett 2011 8 063507 10.1063/1.3624472

[B30] Jeong HY Kim YI Lee JY Choi SY A low-temperature-grown TiO_2_-based device for the flexible stacked RRAM application Nanotechnology 2010 8 115203 10.1088/0957-4484/21/11/115203 20173248

